# Protection from liver-stage malaria is dependent on a fine balance between the number of infected hepatocytes and effector CD8^+^ T cells

**DOI:** 10.1186/1475-2875-13-S1-P83

**Published:** 2014-09-22

**Authors:** Alexandra Spencer, Rhea Longley, Anita Gola, Teresa Lambe, Adrian Hill

**Affiliations:** 1 University of Oxford, Oxford, UK

## Background

Since the demonstration of sterile protection afforded by injection of irradiated sporozoites, CD8^+^ T cells have been shown to play a significant role in protection from liver-stage malaria. This is, however, dependent on the presence of an extremely high number of circulating effector cells, thought to be necessary to scan, locate and kill infected hepatocytes in the short time that parasites are present in the liver. To this day very little is known about the T cell response to malaria in the liver, therefore we have utilized an adoptive transfer system in mice to elucidate the kinetics of CD8^+^ T cell mediated protection following sporozoite challenge.

## Materials and methods

Balb/c mice were immunized with viral vectors expressing the immunodominant epitope from *P. berghei* CS protein, Pb9, to induce high numbers of antigen specific CD8^+^ T cells that were transferred into recipient mice. By CFSE labeling cells we were able to track CD8^+^ T cell migration into the liver and recruitment of cells into division by flow cytometry in the days following sporozoite challenge.

## Results and conclusions

To our surprise we did not detect active recruitment of CD8^+^ T cells into the liver or liver draining lymph nodes in the early days following sporozoite challenge. Cell division was also not required for protection as divided cells were only detected 3 days after challenge. In addition CD8^+^ T cells were shown to only require 24 hours to locate and kill infected hepatocytes. By titrating the number of transferred cells and sporozoites we discovered a threshold of CD8^+^ T cell mediated protection dependent on both the number of infected hepatocytes and circulating CD8^+^ T cells. When mice were challenged with only a small number of sporozoites, not enough CD8^+^ T cells were reactivated, while injection of higher numbers of sporozoites led to better survival. In the absence of detectable changes in the CD8^+^ T cell response at the macroscopic level, we are currently establishing a live-imaging model to study the interplay between effector CD8^+^ T cells and sporozoites in the liver.

